# Supplementary material for: Cardiac Function Evaluation after SARS-CoV-2 mRNA Vaccination in Children and Adolescents: A Prospective Speckle-Tracking Echocardiography Study
Source: Vaccines (Basel). 2023 Aug 9;11(8):1348. doi: 10.3390/vaccines11081348 (PMC10458879; doi:10.3390/vaccines11081348)
Supplement: Supplementary file 1 [file vaccines-11-01348-s001.zip › vaccines-2502831-supplementary.pdf]

Supplemental materials

Material and Methods

Table S1. COVID-19 vaccines delivery timeline in the European and Italian pediatric population updated up to the end of June 2020 (end of the study).

Type of vaccine	Age group	EMA* authorization	AIFA* authorization	Primary regimen schedule and doses	Extension of indication for use as a booster approved by AIFA
**Cominarty (BioNTech/Pfizer's vaccine)**	16 and older	December, 21th, 2020[1]	December, 22th, 2020[2]	30 mcg/dose, 2 doses cycle 21 days away from each other	December, 6th, 2021; 30 mcg/dose, independently from the previous type of vaccine administered, at least 4 months away from the completion of the primary cycle[3]
**Cominarty (BioNTech/Pfizer's vaccine)**	12-15 years old	May, 28 th, 2021[4]	June, 4 th, 2021[5]	30 mcg/dose, 2 doses cycle 21 days away from each other	January, 5 th, 2022; 30 mcg/dose, independently from the previous type of vaccine administered, at least 4 months away from the completion of the primary cycle[6]
**Spikevax (previously**	12-17 years old	July, 23 th, 2021[7]	July, 29 th, 2021[8]	100 mcg/dose, 2 doses	Not approved

**COVID-19 vaccine Moderna)**				cycle 28 days away from each other	
**Cominarty (BioNTech/Pfizer's vaccine)**	5-11 years old	November, 25 th, 2021[9]	December 1 st, 2021[10]	10 mcg/dose, 2 doses cycle 21 days away from each other	Not approved
**Spikevax (previously COVID-19 vaccine Moderna)**	5-11 years old	Not approved	Not approved		Not approved

*European Medicines Agency (EMA); Agenzia Italiana del Farmaco (AIFA);

References

1 First COVID-19 vaccine approved for children aged 12 to 15 in EU | European Medicines Agency, available at: https://www.ema.europa.eu/en/medicines/human/EPAR/comirnaty#:~:text=Comirnaty%20received%20a%20conditional%20marketing,authorisation%20on%2010%20October%202022.

2 La Commissione Tecnico Scientifica di AIFA (CTS) approva il vaccino BioNTech/Pfizer. Comunicato AIFA n. 620 22 dicembre 2020, available at https://www.aifa.gov.it/documents/20142/0/Comunicato%20AIFA%20n.%20620%20-%20Autorizzato%20il%20vaccino%20BioNTech%20Pfizer.%20Sul%20sito%20AIFA%20risposte%20alle%20domande%20pi%C3%B9%20frequenti(1).pdf

3 La Commissione Tecnico Scientifica di AIFA (CTS) approva la dose booster con il vaccino Comirnaty per la fascia di età a partire dai 16 anni. Available at https://www.trovanorme.salute.gov.it/norme/renderNormsanPdf?anno=2021&codLeg=84077&parte=1%20&serie=null

4 First COVID-19 vaccine approved for children aged 12 to 15 in EU | European Medicines Agency. https://www.ema.europa.eu/en/news/first-covid-19-vaccine-approved-children-aged-12-15-eu

5 La Commissione Tecnico Scientifica di AIFA (CTS) approva l'utilizzo del vaccino Comirnaty per la fascia di età 12-15 anni. Comunicato AIFA n. 647 31 maggio 2021, available at https://www.aifa.gov.it/documents/20142/1289678/Comunicato_stampa_AIFA_n.647.pdf

6 La Commissione Tecnico Scientifica di AIFA (CTS) approva la dose booster con il vaccino Comirnaty per la fascia di età 12-15 anni. Comunicato AIFA n. 681 5 gennaio 2022, available at https://www.aifa.gov.it/documents/20142/1618244/Comunicato_AIFA_N.681.pdf

7 COVID-19 vaccine Spikevax approved for children aged 12 to 17 in EU | European Medicines Agency. Accessed February 4, 2022 https://www.ema.europa.eu/en/news/covid-19-vaccine-spikevax-approved-children-aged-12-17-eu
8 La Commissione Tecnico Scientifica di AIFA (CTS) approva l'utilizzo del vaccino Spikevax (Moderna) per la fascia di età 12-17 anni. Comunicato AIFA n. 656 28 luglio 2021, available at https://www.aifa.gov.it/documents/20142/1289678/Comunicato_AIFA_656.pdf
9 Comirnaty COVID-19 vaccine: EMA recommends approval for children aged 5 to 11 | European Medicines Agency. Accessed February 4, 2022. https://www.ema.europa.eu/en/news/comirnaty-covid-19-vaccine-ema-recommends-approval-children-aged-5-11
10 La Commissione Tecnico Scientifica di AIFA (CTS) approva il vaccino Comirnaty per la fascia di età 5-11 anni. Comunicato AIFA n. 674 1 dicembre 2021, available at https://www.aifa.gov.it/documents/20142/1289678/Comunicato_AIFA_674.pdf
